# Limited contribution of health behaviours to expanding income-related chronic disease disparities based on a nationwide cross-sectional study in China

**DOI:** 10.1038/s41598-018-30256-5

**Published:** 2018-08-21

**Authors:** Qing Wang, Jay J. Shen, Kaitlyn Frakes

**Affiliations:** 1 School of business, Dalian University of Technology, Panjin, 124221 Liaoning China; 2 School of public health, Shandong University, Jinan, 250100 shandong China; 3 Department of Health Care Administration and Policy, School of Community Health Sciences, University of Nevada Las Vegas, 4505 Maryland Parkway, Las Vegas, NV 89154-3023 USA

## Abstract

This study estimated the association of income and prevalence of cardiovascular diseases (CVD) and hypertension, and then quantified the contribution of health behaviors to the association in China. Using the 2013 survey of the China Health and Retirement Longitudinal Study (CHARLS), a logit model was applied to examine income-related health disparities in relation to CVD and hypertension. A four-step regression method was then constructed to measure the role of health behaviors in income-related health disparities. Using indirect effects, mediation by health behaviors was examined. Income-related health disparities in chronic diseases were found to exist in China. Specifically, individuals in the high-income group had a 14% (OR = 0.86; 95% CI 0.73–1.02) and 14% (OR = 0.86; 95% CI 0.76–0.97) lower odds of suffering from CVD and hypertension than those in the low-income group. However, limited evidence shows this association was mediated by health behaviors. The Heaviness of Smoking Index (HSI), heavy drinking, irregular eating, and nap time did not significantly mediate the association of income and prevalence of CVD and hypertension. To curb the rising prevalence of CVD and hypertension in China, policies should focus on the low-income subpopulation. However, healthy behaviors interventions targeting smoking, heavy drinking, unhealthy napping and irregular eating habits among low-income people may be ineffective in reduction of income-related disparities in prevalence of CVD and hypertension.

## Introduction

In the past few decades, China has undergone demographic and epidemiologic transitions characterized by a shift from a predominance of nutritional deficiencies and infectious diseases to those classified as chronic diseases, including cardiovascular diseases (CVD) and hypertension^1^. The number of patients with CVD rose substantially from around 15.7 million in 2010, to 25 million in 2016^2,3^. A long-term upward tendency of the proportion of patients with hypertension was also observed. In 1960, 30 million people had hypertension, however this number increased to 59 million in 1980, and 94 million in 1990^4^. In 2016, there were 270 million Chinese adults with hypertension^2^.

The rise in the prevalence of chronic diseases can arguably be attributed to the complex interaction between increased income and health behaviors^5,6^. According to a global brief on hypertension by the World Health Organization (WHO), social determinants of health, e.g. income, have an impact on behavioral risk factors and in this way, influence the development of chronic disease^7^.

The association between income and chronic disease is well established in high-income countries, with income was negatively related to prevalence of CVD and hypertension. However it is not clear in lower-middle-income countries^8–10^. The association tended to be positive in some lower-middle-income countries (e.g. Vietnam, South Africa and Serbia) but inverse in others (e.g. Ghana, Jamaica)^11–14^.

There are a limited number of studies that examine income-related CVD and hypertension disparities in China. Most of these studies were conducted five or more years ago, and the results were heterogeneous. People with lower-income were usually found to have higher levels of CVD and hypertension^15–19^, however, exceptions were observed. Income was found to be positively associated with CVD and hypertension in the 1990s, when China began to experience epidemiologic transitions^20–22^.

The relationship between lower-income individuals and the increasing prevalence of chronic disease in high-income countries (e.g., the United States, Austria, Canada, Finland, United kingdom, Poland, Portugal, Sweden, and Denmark) may be partially attributed to the increased prevalence of unhealthy behaviors in lower-income groups^23,24^. Lower-income individuals are more likely to live with multiple health risks and are often associated with higher rates of smoking, drinking, lack of adequate physical activity, and sleep^25–27^. However, the empirical results vary greatly^28–47^. The role of health behaviors differed substantially by country. For example, evidence shows that drinking contributes little to the increase in income-related health disparities in England and Spain^40^, whereas drinking exerts a major impact in Denmark^34^.

Little is known about the effects of health behaviors on income-related health disparities in China. Health behaviors amongst income levels were proven to be complex in China. Data from the 2013 survey of the China Health and Retirement Longitudinal Study (CHARLS) showed low-income respondents were less likely to smoke but more likely to have unhealthy naptimes and irregular diets^48^. Therefore, this study aims to update the literature regarding income-related CVD and hypertension disparities in China and evaluate the mediator of health behaviors linking income to disparities in CVD and hypertension in China.

## Results

Table 1 presents the demographic characteristics of the study sample in CHARLS 2013. Respondents in the higher-income groups were more likely to have higher educational attainment and be a wage earner. Additionally, they tended to be younger and living in an urban area. Based on unweight and weighted prevalence of CVD and hypertension, a graded association between income and prevalence of CVD and hypertension was documented (weighted prevalence see appendix). Individuals with higher-income were more likely to report healthy naptimes, regular eating, but more likely to smoke. No pattern of heavy drinking was observed.

**Table 1:** Demographic and behavioral characteristics of the study sample by income group.

Characteristics	High Income (n = 3752)	Medium Income (n = 3963)	Low Income (n = 3998)	P-value from Chi-2 test
CVD (%)	12.21	13.18	13.81	0.100
Hypertension (%)	34.67	36.29	40.70	<0.001
Education				<0.001
No formal education (%)	19.35	27.00	37.74	
No formal education but can read/write (%)	14.82	19.58	21.79	
Elementary school (%)	21.35	23.72	21.74	
Primary school and above (%)	44.48	29.70	18.73	
Employment status				<0.001
Unemployed (%)	16.70	14.68	22.43	
Retired (%)	13.47	10.07	8.46	
Self-employed and farmers (%)	47.01	60.59	63.48	
Wage-earner (%)	22.81	14.66	5.63	
Urban residing (%)	49.87	33.81	23.41	0.001
Age# (year)	57.28 (9.02)	58.51 (8.82)	61.97 (9.57)	<0.001
Male (%)	47.44	47.56	46.82	0.778
Unmarried (%)	7.46	11.05	17.83	0.001
HSI				<0.001
Non-smokers (%)	65.44	66.38	68.57	
Low HSI (%)	11.80	12.73	12.08	
Medial/high HSI (%)	22.76	20.89	19.35	
Heavy drinking (%)	14.39	14.26	13.86	0.779
Irregular meals#	11.57	15.92	16.76	<0.001
Naptime				<0.001
<10 min (%)	52.50	54.32	55.26	
[10–60 min] (%)	33.40	31.77	28.81	
60 min and over (%)	14.10	13.90	15.93	
Over-weight (%)	35.42	30.43	27.89	<0.001

Descriptive statistics of the study sample in CHARLS 2013.

^#^Mean, SD (numbers in the parentheses indicate SD); Cardiovascular diseases: CVD; HSI: the Heaviness of Smoking Index.

Results on the relationship between income and CVD and the mediating role of health behaviors are presented in Table 2. Logit regression analysis indicated that compared to the low-income group, the high-income group was associated with a 14% (OR = 0.86; 95% CI 0.73–1.02) decrease in the odds ratio of CVD.

**Table 2:** Relationship between income and CVD prevalence mediated by health behaviors.

Relationship between income and CVD mediated by HSI		Relationship between income and CVD mediated by heavy drinking		Relationship between income and CVD mediated by eating		Relationship between income and CVD mediated by nap time	
**Step 1 Income-related health disparity in CVD prevalence without adjusting for health behaviors (outcome variable: CVD prevalence)**							
	OR (95% CI)		OR (95% CI)		OR (95% CI)		OR (95% CI)
High income	0.86* (0.73–1.02)	High income	0.86* (0.73–1.02)	High income	0.86* (0.73–1.02)	High income	0.86* (0.73–1.02)
Prob > chi2	0.001	Prob > chi2	0.001	Prob > chi2	0.001	Prob > chi2	0.001
**Step 2a Relationship between income and health behaviors (outcome variable: health behavior)**							
	OR (95% CI% CI)		OR (95% CI)		OR (95% CI)		RRR (95% CI)
High income	1.31*** (1.14–1.52)	High income	0.95 (0.80–1.13)	High income	0.81** (0.68–0.95)	High income^a^	0.95 (0.83–1.07)
						High income^b^	0.85* (0.71–1.00)
Prob > chi2	0.001	Prob > chi2	0.001	Prob > chi2	0.001	Prob > chi2	0.001
**Step 2b Relationship between health behaviors and CVD prevalence (outcome variable: CVD prevalence)**							
	OR (95% CI)		OR (95% CI)		OR (95% CI)		OR (95% CI)
Low HSI	0.96 (0.75–1.23)	Heavy drinking	0.71*** (0.57–0.87)	Irregular eating	1.18* (0.97–1.44)	Nap time < 10 mins	1.15** (1.00–1.33)
Medial/high HSI	1.39*** (1.15–1.67)					>60 mins	1.15 (0.97–1.38)
Prob > chi2	0.001	Prob > chi2	0.001	Prob > chi2	0.001	Prob > chi2	0.001
**Step 3 Income-related health disparity in CVD prevalence adjusting for health behaviors (outcome variable: CVD prevalence)**							
	OR (95% CI)		OR (95% CI)		OR (95% CI)		OR (95% CI)
High income	0.86* (0.72–1.03)	High income	0.85* (0.72–1.02)	High income	0.86* (0.72–0.97)	High income	0.86* (0.73–1.03)
Low HSI	0.96 (0.75–1.23)	Heavy drinking	0.70*** (0.57–0.87)	Irregular eating	1.17 (0.96–1.44)	Nap time < 10 mins	1.15* (1.00–1.33)
Medial/high HSI	1.40*** (1.16–1.68)					>60 mins	1.15 (0.97–1.38)
Prob > chi2	0.001	Prob > chi2	0.001	Prob > chi2	0.001	Prob > chi2	0.001
**Mediation statistics**							
	Effect (Bias-corrected 95% CI)		Effect (Bias-corrected 95% CI)		Effect (Bias-corrected 95% CI)		Effect (Bias-corrected 95% CI)
Indirect effect	−0.001 (−0.002–0.000)	Indirect effect	−0.001 (−0.004–0.002)	Indirect effect	−0.001 (−0.003–0.002)	Indirect effect	0.000 (−0.001–0.001)
Total effect	−0.026 (−0.057–0.004)	Total effect	−0.032 (−0.060–0.002)	Total effect	−0.032 (−0.057–0.006)	Total effect	−0.308 (−0.061–0.001)

^a^Outcome variable: nap time below 10 mins.

^b^Outcome variable: nap time above 60 mins.

***p < 0.01, **p < 0.05, *p < 0.1.

The high-income group had an odds of medial/high Heaviness of Smoking Index (HSI) versus the combined group of low HSI and non-smokers that is 1.31 times that of the low-income group (OR = 1.31; 95% CI 1.14–1.52), and the medial or high HSI smokers had a 39% (OR = 1.39; 95% CI 1.15–1.67) higher odds of suffering from CVD than non-smokers. The association of income and CVD were very similar before and after adjusting for HSI (OR = 0.86; 95% CI 0.72–1.03) and the indirect effect was close to zero.

Income was not related to heavy drinking (OR = 0.95; 95% CI 0.80–1.13). As a result, it was concluded that heavy drinking was not associated with income-related health disparities in CVD.

Compared to the low-income group, those with high-income were less likely to report irregular eating by 19% (OR = 0.81; 95% CI 0.68–0.95), and irregular eating was associated with an increased odds of CVD (OR = 1.18; 95% CI 0.97–1.44). However, with income and HSI controlled, the association of income and CVD was very similar to those without adjusting for irregular eating. Thus, there was limited evidence of irregular eating mediating income-related health disparities.

Income did not affect prevalence of short nap times (OR = 0.95; 95% CI 0.83–1.07), and shorter nap times were not associated with income-related CVD disparities. Those with high-income were less likely to report longer nap times than low-income respondents by 15% (OR = 0.85; 95% CI 0.71–1.00). Since longer nap times were not associated with CVD, longer nap times didn’t mediate income-related CVD disparities.

Table 3 shows the relationship between income and hypertension and the mediating role of health behaviors. Income-related health disparities in hypertension were found to exist in China. Individuals in the high-income group had an 14% (OR = 0.86; 95% CI 0.76–0.97) lower odds of suffering from hypertension than those in the low-income group.

**Table 3:** Relationship between income and hypertension prevalence mediated by health behaviors.

Relationship between income and hypertension mediated by HSI		Relationship between income and hypertension mediated by heavy drinking		Relationship between income and hypertension mediated by eating		Relationship between income and hypertension mediated by nap time	
**Step 1 Income-related health disparity in hypertension prevalence without adjusting for health behaviors (outcome variable: hypertension prevalence)**							
	OR (95% CI)		OR (95% CI)		OR (95% CI)		OR (95% CI)
High income	0.86** (0.76–0.97)	High income	0.86** (0.76–0.97)	High income	0.86** (0.76–0.97)	High income	0.86** (0.76–0.97)
Prob > chi2	0.001	Prob > chi2	0.001	Prob > chi2	0.001	Prob > chi2	0.001
**Step 2a Relationship between income and health behaviors (outcome variable: health behavior)**							
	OR (95% CI)		OR (95% CI)		OR (95% CI)		RRR (95% CI)
High income	1.31*** (1.14–1.52)	High income	0.95 (0.80–1.13)	High income	0.81** (0.68–0.95)	High income^a^	0.95 (0.83–1.07)
						High income^b^	0.85* (0.71–1.00)
Prob > chi2	0.001	Prob > chi2	0.001	Prob > chi2	0.001	Prob > chi2	0.001
**Step 2b Relationship between health behaviors and hypertension prevalence (outcome variable: hypertension prevalence)**							
	OR (95% CI)		OR (95% CI)		OR (95% CI)		OR (95% CI)
Low HSI	1.11 (0.93–1.33)	Heavy drinking	1.20** (1.03–1.39)	Irregular eating	0.97 (0.83–1.13)	Nap time < 10 mins	1.16** (1.03–1.30)
Medial/high HSI	1.06 (0.92–1.23)					>60 mins	1.26*** (1.10–1.43)
Prob > chi2	0.001	Prob > chi2	0.001	Prob > chi2	0.001	Prob > chi2	0.001
**Step 3 Income-related health disparity in hypertension prevalence adjusting for health behaviors (outcome variable: hypertension prevalence)**							
	OR (95% CI)		OR (95% CI)		OR (95% CI)		OR (95% CI)
High income	0.86** (0.76–0.97)	High income	0.86** (0.76–0.97)	High income	0.86** (0.76–0.97)	High income	0.86** (0.76–0.97)
Low HSI	1.11 (0.93–1.33)	Heavy drinking	1.19* (1.03–1.39)	Irregular eating	0.96 (0.83–1.12)	Nap time < 10 mins	1.16 (1.03–1.30)
Medial/high HSI	1.07 (0.93–1.24)					>60 mins	1.26*** (1.10–1.42)
Prob > chi2	0.001	Prob > chi2	0.001	Prob > chi2	0.001	Prob > chi2	0.001
**Mediation statistics**							
	Effect (Bias-corrected 95% CI)		Effect (Bias-corrected 95% CI)		Effect (Bias-corrected 95% CI)		Effect (Bias-corrected 95% CI)
Indirect effect	−0.001 (−0.002–0.000)	Indirect effect	−0.000 (−0.001–0.000)	Indirect effect	0.002 (−0.001–0.004)	Indirect effect	0.000 (−0.001–0.001)
Total effect	−0.057 (−0.078–0.036)	Total effect	−0.052 (−0.078–0.038)	Total effect	−0.057 (−0.078–0.037)	Total effect	−0.058 (−0.080–0.036)

^a^Outcome variable: nap time below 10 mins.

^b^Outcome variable: nap time above 60 mins.

***p < 0.01, **p < 0.05, *p < 0.1.

HSI and irregular eating were not significantly associated with hypertension after adjusting for those obese and overweight (Low HSI: OR = 1.11; 95% CI 0.93–1.33; High/Medial HSI: OR = 1.06; 95% CI 0.92–1.23; irregular eating: OR = 0.97; 95% CI 0.83–1.13). As a result, HSI and irregular eating did not play a role in income-related health disparities measured by hypertension prevalence.

Income didn’t influence heavy drinking (OR = 0.95; 95% CI 0.80–1.13) and shorter nap times (OR = 0.95; 95% CI 0.83–1.07), thus, heavy drinking and shorter nap times were not mediators of income-related health disparities in hypertension.

Those with high-income were less likely to report longer nap times than low-income respondents by 15% (OR = 0.85; 95% CI 0.71–1.00), longer nap times were associated with an increased odds of hypertension (OR = 1.26; 95% CI 1.10–1.43). Nevertheless, the effects of income on prevalence of hypertension before and after adjustment of income and nap times were the same, thus, longer nap times were not associated with income-related CVD disparities.

## Discussion

Using a nationwide survey, researchers conducted a cross-sectional survey in a large representative sample among the Chinese middle-aged and elderly population. This study quantifies the contribution of health behaviors to the relationship between income and prevalence of chronic disease measured by CVD and hypertension. Our study shows the existence of wide income inequalities regarding the risks of CVD and hypertension. These results also suggest that health behaviors may not solely explain these inequalities.

The distribution of CVD and hypertension was substantially patterned by income. Individuals with lower-income generally showed a higher prevalence of CVD and hypertension. This pattern has been frequently observed in high-income countries, but is contradictory to Vietnam, South Africa and Serbia^8–14^. A possible explanation is the association changes along the epidemiological transition^10^. The prevalence of chronic disease seemed to be greater in the higher-income group in populations from countries (e.g. Vietnam, South Africa and Serbia) at an early stage of the epidemiologic transition, whereas the prevalence was lower in higher-income categories in populations from countries (e.g. America) at an advanced stage of the epidemiologic transition^8–14^. In China, chronic diseases were once known as “diseases of the rich” (fugui bing) in the 1990s, when China started its epidemiological transition. Studies that examine this time period found that income was positively associated with CVD and hypertension^21,22^. However, as the epidemiological transition progressed, low-income individuals are found to be more vulnerable to chronic diseases presently^49^. Consequently, strategies focused on preventing and controlling modifiable risk factors should be considered, especially with attention to populations with low-income.

Some studies insisted that adherence to health behaviors may substantially lower the burden of chronic disease in the Chinese population. Additionally, compared to the high-income group, individuals with low-income were more likely to have some risk factors relating to some health behaviors in China^50–54^. Thus, although no study estimates the role of health behaviors between income and risk of chronic disease, it is assumed that prompting health behaviors may not only control prevalence of chronic diseases but also decrease income-related risks of chronic disease.

Our study exclusively evaluated the role of smoking, drinking, eating, and nap time pertaining to income-related health disparities among the Chinese population. Nonetheless, limited evidence was found in our study. Nap time, HSI, irregular eating and heavy drinking did not impact the income-related health disparities in CVD and hypertension, whereas health behaviors partially explained the income-related health disparities in some western societies. The results vary, possibly due to the characteristics of health behaviors. First, health behaviors may not be related to health outcomes. Although irregular eating showed income-related disparities in our data, irregular eating did not influence the prevalence of hypertension after adjusting for those obese and overweight.

Second, countries with smaller differences in health behaviors may have less income-related health disparities in CVD and hypertension^45^. For instance, disparities in mortality in Italy and Spain are smaller than in other European countries. This has proved to be attributed to socioeconomic status differences in smoking behaviors^55,56^. In China, heavy drinking across income levels was not be observed, subsequently, drinking was not found to be associated with income-related health disparities.

Our study has some limitations. First, we did not figure out the determinants of income-related health disparities in CVD and hypertension. Second, due to data constraints, important health behaviors (e.g., physical activity) that may explain morbidity were not included, which may lead to variation in our results from previous literature. Therefore, the role of health behaviors in the relationship between income and prevalence of CVD and hypertension can only be concluded with caution. Third, there may be reporting bias of suffering from CVD and hypertension due to diagnostics, especially in less-developed communities or provinces^57^. The bias may lead to tendentious estimations regarding the role of health behaviors.

Aside from the limitations, the study may still have some policy implications. The Chinese government makes great efforts to control the increasing prevalence of chronic disease^2,3^. The existing interventions focus on populations with low-income, which is consistent with our results^2–5,15–17^. A targeted health intervention could lead to a favorable impact due to higher prevalence of CVD and hypertension among the lower-income group.

However, the policies that try to control income-related health disparities in chronic disease by focusing on unhealthy behaviors among low-income individuals may be ineffective. Evidence suggests that health behaviors such as smoking, drinking, napping and regular eating habits may not shape the income pattern of CVD and hypertension. Therefore, policies on reduction of income-related disparities in health may not be restricted to health behaviors targeting smoking, drinking, napping and regular eating habits and may consider other risk factors.

This does not mean interventions focused on improving health behaviors should be discarded. According to the WHO, four of nine targets for prevention and control of chronic diseases to be attained by the year 2025 were related to health behaviors (tobacco use, harmful use of alcohol)^58^. Health behaviors such as smoking, drinking, eating, physical activity and nap times are recommended for health promotion in China, too^2,3^. Our results found that HSI, heavy drinking, irregular eating and napping were significantly related to the prevalence of CVD, while drinking and napping were associated with prevalence of hypertension significantly. However, previous policies focus on interventions targeting health behaviors without take chronic disease characteristics into consideration^2,3^. Considering the heterogeneous association of health behaviors and chronic disease, we recommend future policies aimed at improving behaviors may be tailored specifically for different chronic diseases and their characteristics.

## Methods

### Participants

The sample chosen for this study is from the 2013 survey of CHARLS^34^. CHARLS is a nationally representative sample of people aged 45 and older, and investigates a broad range of areas, including demographic background, family structure, work status, retirement and pension, income, and health information for mainland China. Individuals aged 45 or older and their spouses were interviewed face-to-face in each household. The response rate of the survey was over 80%. Details of the data have been previously described^59^. The survey was approved by the Institutional Review Board of Peking University (IRB00001052-11014). All methods were performed in accordance with the relevant guidelines and regulations. All respondents in the study gave informed consent.

Using multi-stage stratified probability-proportionate-to-size sampling, the sample in CHARLS belonged to approximately 10,000 households in 150 counties/districts (a total of 450 villages/resident communities). First, 150 county-level units from 28 provinces were chosen. Then, three primary sampling units – administrative villages (*cun*) in rural areas and neighborhoods (*shequ*) in urban areas – were then selected from each county-level unit. All of the dwellings in each primary sampling unit were outlined on Google Earth maps with the “CHARLS- GIS” software package, which was specifically designed for the survey. Finally, 24 of the mapped households in each primary sampling unit were randomly selected. One member aged 45 years or older and his or her spouse were randomly chosen as subjects of the survey. Overall, biomarker samples were collected from 12,957 individuals by medical staff. A total of 251 individuals who were less than 45 years old were excluded from the analysis. After excluding the observations with missing values, the final sample size was 11,713 in the cross-sectional data.

### Variables

#### Income

CHARLS includes several income questions to capture sources of household income extensively. The income questions pertain to labor earnings (e.g., wages and salaries or self-employment income), non-labor income (e.g., interest, dividends, or rental income), and private transfer income (e.g., alimony or workers’ compensation and public transfer income such as unemployment, welfare, social security). Irregular income, such from stock options and capital gains, was not considered. The resources available to any family member not only depend on the family’s income, but also the number of family members sharing that income. Therefore, household-income per capita was used to measure income in the study. Family size was defined as a census subfamily, which includes all related individuals in a household. Accordingly, income was adjusted for family size by dividing it by the square root of the number of family members. This adjustment closely matches the adjustment for family size used by the U.S. Census Bureau’s poverty thresholds^60^. Subsequently, household-income per capita was ranked and divided into three tertiles (i.e., high, medium and low), with the low group as reference.

#### Health behaviors

Nicotine dependence, heavy drinking, eating irregular meals, and unhealthy napping, which are well-identified modifiable risk factors of CVD and hypertension, were concluded to measure health behaviors^50^. Also, the burden of behavioral risk factors is affected by income^8^. In CHARLS, the HSI measures nicotine dependence. The HSI score is based on two items, cigarettes smoked per day and time to first cigarette after waking up, both taking values from 0 to 3. These two items are then totaled, with a score from 0 to 6^61^. ‘Cigarettes smoked per day’, was based on the responses of the CHARLS question ‘How many cigarettes do you smoke each day now?’ ‘Cigarettes smoked per day’ was recoded into an ordinal variable from 0 to 3 representing the level of smoking (0 = smoked 0 to 9 cigarettes per day, 1 = 10 to 19 cigarettes per day, 2 = 20 to 29 cigarettes per day, 3 = more than 30 cigarettes per day). Time to first cigarette after waking up was classified into 4 groups with 0 = more than 1 hour, 1 = within 31 to 60 minutes, 2 = with 6 to 30 minutes and 3 = within 5 minutes. HSI was then classified into a three-category variable: non-smokers (HSI = 0), low HSI (HSI = 1 OR 2), and medial/high HSI (HSI > = 3)^62^.

Respondents were asked if they drank beer or any other alcoholic beverage in the last 12 months. Those who responded in the affirmative were asked further questions on the type of beverage (beer, grape wine, liquor) and the typical amount of alcoholic drinks they consumed on a single occasion (reported by number of bottles (640 ml) for beer and number of liang (50 grams) for wines and liquor), assuming the following alcohol content by volume (v/v) to be typical in China, beer 4%, grape wine 12%, liquor 45%^63,64^. Heavy drinking episodes were classified as the consumption of more than 60 grams of alcohol on one occasion for men, and more than 40 grams for women^65^.

In China, the general public normally eats three meals a day, thus respondents who ate 2 meals daily and below were categorized as people who ate irregular meals^66^.

Napping is well accepted and practiced by many Chinese persons^67^. 10 to 60 minute naps were considered healthy naps and others were categorized as unhealthy naps^68,69^. In CHARLS, respondents were asked “How long did you take a nap after lunch during the past month”? We classified napping times into three groups (below 10 minutes, 10 to 60 minutes and above 60 minutes with 10 to 60 minutes as the reference group).

#### CVD and hypertension

Our primary outcomes were the prevalence of CVD and hypertension. The diagnosis of CVD and hypertension was based on the self-reported diagnosis by a doctor based on the CHARLS question, “Have you ever been diagnosed with hypertension, heart attack, coronary heart disease, angina, congestive hear failure, stroke, or other heart problem by a doctor”?

In addition, for the undiagnosed cases of hypertension, the clinical test in CHARLS was used to measure the diagnosis. Respondents’ blood pressure was measured three times. The means of systolic and diastolic measurements were respectively calculated, and a variable for being hypertensive was constructed. In accordance with cutoffs for hypertension provided by the WHO, if the mean systolic was 140 or above or the mean diastolic was 90 or above, the case was considered hypertensive for the purpose of this study^7^.

#### Demographic and other measures

Demographic variables included age, gender (reference group, female), and marital status (married was encoded as 0 for separated, divorced, widowed, never married and 1 for cohabitated. Married group was the reference). Education, employment status, and urban residing measured socioeconomic status. Educational attainment was defined according to four levels, no formal education, no formal education but can read and write, elementary school, and primary school or above. Three dummy variables were created for educational attainment, with no formal education serving as the reference group. Employment status was classified as unemployed, retired, self-employed, and wage earner, with unemployment serving as the reference group. Being overweight (according to the Asian cutoffs provided by the WHO, overweight constitutes a person with a Body Mass Index greater than or equal to 23) and outpatient utilization in the last month were also controlled^70^.

### Statistical methods

We first carried out a descriptive analysis for each income group. P-values were calculated by using the Chi-square test for categorical variables, and one-way ANOVA for continuous variables between income groups. Weighted prevalence of CVD and hypertension by income was also calculated adjusting for sample design (See appendix).

**Figure 1:**
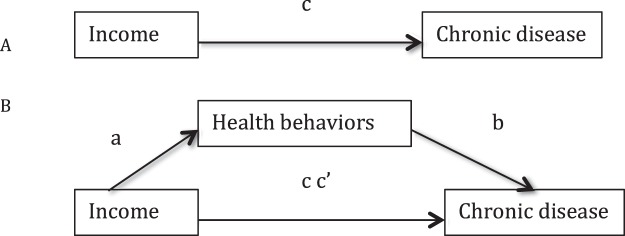
(**A**) Direct relationships between income and chronic disease. (**B**) Relationships between between income and chronic disease mediated by health behaviors.

Subsequently, the hypothesis that any association between income and chronic disease was mediated by health behaviors, was examined using the four-step regression approach which is summarized in Fig. 1A,B^71^. The four-step method involves testing a direct path between the exposure and the outcome and then estimating by how much the association is reduced by the inclusion of the potential mediator. To be specific, (1) the logit model was constructed to test whether three-level income significantly affects the prevalence of CVD and hypertension without adjusting for health behaviors (c); (2) income significantly affects health behaviors (a). For heavy drinking and irregular eating, HSI, and nap time, logit model, ordered logit model and multinomial logit model were used, respectively; (3) the logit model was applied to test whether health behaviors have a significant unique effect on health outcomes (b); (4) the effect of three-level income on health outcomes lessens upon the addition of health behaviors to the model (cc′). Odd ratios (OR) and 95% Confidence intervals (CI) were reported for logit and ordered logit models. Relative risk ratios (RRR) and 95% Confidence intervals (CI) were reported for multinomial logit model. Demographic socioeconomic status and health status were controlled in all the models (a, b, c and cc′). All regression models were weighted using sample weights to correct for the multistage stratified sampling design. Mediation statistics, including the indirect effect and total effect, were obtained using a modified version of the user-written Stata “binary_mediation” command. In addition, bias-adjusted 95% confidence intervals were derived using bootstrapping methods. STATA 14 was used for all calculations.

### Availability of data and materials

The dataset supporting the conclusions of this article is available in http//charls.pku.edu.cn/en.

### Ethical approval and consent to participate

The CHARLS survey used was approved by the Institutional Review Board of Peking University. All the respondents provided consent to participate in this survey.

## Electronic supplementary material

Weighted prevalence of CVD and hypertension by income group
