# Supplementary material for: Limited contribution of health behaviours to expanding income-related chronic disease disparities based on a nationwide cross-sectional study in China
Source: Sci Rep. 2018 Aug 21;8:12485. doi: 10.1038/s41598-018-30256-5 (PMC6104030; doi:10.1038/s41598-018-30256-5)
Supplement: Supplementary file 1 — Weighted prevalence of CVD and hypertension by income group [file 41598_2018_30256_MOESM1_ESM.pdf]

Income-related health disparities in chronic diseases and the role of health behaviors, Evidence from China

Qing Wang, Ph.D. [a],* Jay J. Shen, PhD [b], Kaitlyn Frakes, B.S.[c]

[a] School of business, Dalian University of Technology, Panjin, 124221, Liaoning, China.

Corresponding author. E-mail address, wangqing1984@126.com. Tel, 8618811795046.

[b] Department of Health Care Administration and Policy, School of Community Health Sciences, University of Nevada Las Vegas. 4505 Maryland Parkway, Las Vegas, NV 89154-3023, USA. jay.shen@unlv.edu

[c] Department of Health Care Administration and Policy, School of Community Health Sciences, University of Nevada Las Vegas. 4505 Maryland Parkway, Las Vegas, NV 89154-3023, USA. frakek1@unlv.nevada.edu

Appendix table 1 Weighted prevalence of CVD and hypertension by income

Characteristics	High Income	Medium Income	Low Income
CVD	12.35	13.03	13.26
Hypertension	33.40	36.95	40.99
